# Unusual Longevity of Edwards Syndrome: A Case Report

**DOI:** 10.3390/genes11121466

**Published:** 2020-12-07

**Authors:** Abbas Alshami, Steven Douedi, Melissa Guida, Firas Ajam, Dhaval Desai, Vincent Zales, Dawn M Calderon

**Affiliations:** 1 Department of Medicine, Jersey Shore University Medical Center, Neptune, NJ 07753, USA; Abbas.Alshami@hackensackmeridian.org; 2 Department of Biology, Ramapo College of New Jersey, Mahwah, NJ 07430, USA; mguida@ramapo.edu; 3 Department of Cardiology, Jersey Shore University Medical Center, Neptune, NJ 07753, USA; Firas.Ajam@hackensackmeridian.org (F.A.); Dhaval.Desai@hackensackmeridian.org (D.D.); Dawn.Calderon@hackensackmeridian.org (D.MC.); 4 Department of Pediatrics, Jersey Shore University Medical Center, Neptune, NJ 07753, USA; Vincent.Zales@hackensackmeridian.org

**Keywords:** trisomy 18, Edwards syndrome, genetic screening, chromosome, karyotype

## Abstract

Background: Trisomy 18, also known as Edwards syndrome, was first described in the 1960s and is now defined as the second most common trisomy. While this genetic disease has been attributed to nondisjunction during meiosis, the exact mechanism remains unknown. Trisomy 18 is associated with a significantly increased mortality rate of about 5–10% of patients surviving until 1 year of age. We present a case of a 26-year-old female diagnosed with trisomy 18, well outliving her life expectancy, maintaining a stable state of health. Case Presentation: A 26-year-old female with non-mosaic Edwards syndrome presented to the clinic for follow up after recent hospitalization for aspiration pneumonia. The definitive diagnosis of trisomy 18 was made prenatally utilizing chromosomal analysis and G-banding and fluorescence in situ hybridization (FISH) on cells obtained via amniocentesis. Her past medical history is characterized by severe growth and intellectual limitations; recurrent history of infections, especially respiratory system infections; and a ventricular septal defect (VSD) that was never surgically repaired. She remains in good, stable health and is under close follow-up and monitoring. Conclusions: Despite the fact that Edwards syndrome carries a significantly high mortality rate due to several comorbidities, recent literature including this case report has identified patients surviving into adulthood. Advancements in early detection and parent education have likely allowed for these findings. We aim to present a case of an adult with trisomy 18, living in stable condition, with an importance on medical follow-up.

## 1. Introduction

Trisomy 18, or Edwards syndrome, was first described in 1960 and is one of the most common chromosomal disorders, characterized by the presence of an extra chromosome 18, thus also called trisomy 18 [1,2]. Three phenotypes have been described for this syndrome: full, mosaic, or partial trisomy 18, with full trisomy 18 being the most common phenotype [2,3]. Several clinical features have been described for Edwards syndrome, including growth retardation; craniofacial features; and different hands and feet findings, such as clenched fists, underdeveloped thumbs, and club feet [2,4]. In addition, major malformations involving several organ systems, such as cardiovascular, nervous, and genitourinary systems, result in significant morbidity and high mortality among these patients [4,5]. The reported median survival of patients with Edwards syndrome is only 4 days, and only 5–10% of the patients survive until 1 year of age [5,6]. We describe a case of a patient with Edwards syndrome confirmed by karyotype who thus far has survived to 26 years old and is in a stable condition.

## 2. Case Presentation

**Figure 1:**
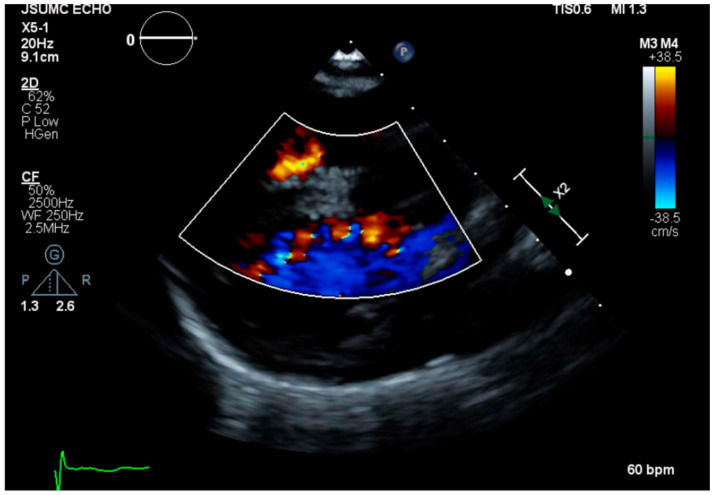
Echocardiogram showing a small muscular ventricular septal defect with left to right shunting.

A 26-year-old woman with non-mosaic Edwards syndrome presented to the clinic for follow up after recent hospitalization for aspiration pneumonia. The definitive diagnosis of trisomy 18 was made prenatally utilizing chromosomal analysis and G-banding and fluorescence in situ hybridization (FISH) on cells obtained via amniocentesis. Her past medical history is characterized by severe growth and intellectual limitations, recurrent history of infections, especially respiratory system infections, and a ventricular septal defect (VSD) that was never surgically repaired. She has been in her usual state of health under the complete care of her mother for all daily living needs for the past seven years until recently, when she was found to be in respiratory distress and hypoxic (O_2_ saturation decreased to 77% on room air). She was admitted to the hospital and was diagnosed with and treated for aspiration pneumonia, from which she recovered and was discharged shortly after with significant improvement of her respiratory symptoms. While this was not her first episode of aspiration pneumonia, she was deemed inappropriate for gastrostomy tube placement during several previous hospitalizations. During the follow-up clinical visit, she was wheelchair bound, cheerful and able to mumble some verbal sounds, and in no acute distress. Her vital signs showed blood pressure of 104/64 mmHg, heart rate of 87 beats per minute, respiratory rate of 16 breaths per minute, and weight of 45 pounds. Physical examination revealed a small head, no occipital protuberance, low-set ears, small jaw, broad nasal bridge, and ocular hypertelorism; however, both vision and auditory acuity were intact. Chest examination revealed severe kyphoscoliosis; clear lungs upon auscultation; and a soft non-holosystolic murmur, best heard at the left lower sternal border. Abdominal examination showed soft abdomen, with positive bowel sounds and no palpable organomegaly. Extremities were well perfused with 2 + distal pulses. The hands were clenched, and palmar creases were normal. No clubbing, cyanosis, or rocker-bottom feet were noted. Echocardiogram was only remarkable for a small muscular VSD with left to right shunting (Figure 1). Laboratory results were unremarkable on follow-up, including any significant hematological, renal, or liver impairment. 

## 3. Discussion

Trisomy 18, or Edwards Syndrome, is due to a chromosomal disorder resulting in an extra copy of chromosome 18, is the second most common trisomy behind trisomy 21, and is found in 1/6000 to 1/8000 live births [2,3,7]. The prevalence of autosomal trisomies increases with advancing maternal age and is diagnosed using serum marker screening and sonography, with G-banded karyotyping being the confirmatory test [3]. It has been suspected that nondisjunction during maternal meiosis or less commonly post zygotic nondisjunction mitosis, leading to mosaicism, is the cause of trisomy 18, yet the exact mechanism remains unknown [3]. Despite a high risk of stillbirths and fetal loss, more than 50% of infants diagnosed with trisomy 18 live beyond 1 week of age; however, only 5–10% will survive beyond 1 year old [3,7,8]. Rarely, patients can survive to adulthood with close monitoring and follow-up. Literature has described patients in their 20 s; however, this is extremely uncommon, making the age of our patient remarkable [9,10].

The substantial multiple major malformations these patients develop can explain the reported high mortality rate. Major causes of death include central apnea, respiratory insufficiency, aspiration, and heart failure due to structural malformations [3,11]. Our patient had a recent episode of aspiration pneumonia along with several in the past requiring hospitalizations; however, it was successfully managed, and the patient remained clinically stable throughout her entire hospital course. Structural cardiac defects represent a major comorbidity in these patients as well. In a case series of 174 fetuses with trisomy 18, more than 70% had abnormal cardiac findings, including VSD, patent ductus arteriosus (PDA), tetralogy of Fallot, and atrioventricular defects [7,11,12]. Our patient has a well-functioning heart and no major malformations other than a small VSD, which potentially could have helped in the patient’s long-term survival. Some more recent studies have shown more favorable outcomes in patients with trisomy 18 and cardiac defect surgery in select populations with significant defects [12]. In addition to cardiac defects, a large percentage of these patients are found to have renal abnormalities, including horseshoe kidney, hydronephrosis, and polycystic kidney disease, as well as central nervous system abnormalities such as Dandy–Walker syndrome [11,13,14]. Almost all patients with trisomies, including trisomy 18, have been found to have developmental delay, as seen in our patient [3,11].

Genetic abnormalities, such as trisomy 18, come at a great risk to both the fetus and mother. Advances in maternal fetal medicine have allowed for more accurate and earlier diagnosis of genetic diseases, including trisomies [15]. Coupled with genetic counseling and education, parents can be equipped with the knowledge and symptoms of these difficult disorders and therefore make informed decisions [15,16]. As advancements in medicine and genetic education continue to progress, we may see an increased survival of patients with trisomy 18 and other genetic syndromes.

## 4. Conclusions

Trisomy 18, also known as Edwards syndrome, was first described in the 1960s and is now defined as the second most common trisomy. With a wide range of symptoms and complications, and a mortality rate of greater than 90% past the age of 1 year old, our patient’s survival to 26 years of age is very remarkable and unusual. Recent advancements in medicine and literature, such as this case report, have defined older individuals living with trisomy 18. With little knowledge of how these individuals survive significantly longer than others, it is important to recognize the comorbidities associated with this genetic disease.
